# ATP Release from Mast Cells by Physical Stimulation: A Putative Early Step in Activation of Acupuncture Points

**DOI:** 10.1155/2013/350949

**Published:** 2013-06-04

**Authors:** Lina Wang, Jacek Sikora, Lei Hu, Xueyong Shen, Ryszard Grygorczyk, Wolfgang Schwarz

**Affiliations:** ^1^Acupuncture and Moxibustion College, Shanghai University of Traditional Chinese Medicine, 1200 Cailun Road, Shanghai 201203, China; ^2^Shanghai Research Center for Acupuncture and Meridians, 199 Guoshoujing Road, Shanghai 201203, China; ^3^Research Centre, Centre Hospitalier de l'Université de Montréal, 3850 St. Urbain Street, Montréal, QC, Canada H2W 1T8; ^4^Department of Biology and Environmental Protection, Poznan University of Medical Sciences, 1/2 Dluga Street, 61-848 Poznan, Poland; ^5^Department of Medicine, Université de Montréal, Montreal, QC, Canada H3C 3T5; ^6^Institute for Biophysics, Goethe-University Frankfurt, Max-von-Laue Stra**β**e 1, 60438 Frankfurt am Main, Germany

## Abstract

In Chinese medicine acupuncture points are treated by physical stimuli to counteract various diseases. These stimuli include mechanical stress as applied during the needle manipulation or tuina, high temperatures as applied during moxibustion, and red laser light applied during laser acupuncture. This study aimed to investigate cellular responses to stimuli that might occur in the tissue of acupuncture points. Since they have a characteristically high density of mast cells that degranulate in response to acupuncture, we asked whether these processes lead to ATP release. We tested in *in vitro* experiments on mast cells of the human mast-cell line HMC-1 the effects of the physical stimuli; mechanical stress was applied by superfusion of the cells with hypotonic solution, heat was applied by incubation of the cells at 52°C, and red laser light of 657 nm was used for irradiation. We demonstrate that all the stimuli induce ATP release from model human mast HMC-1 cells, and this release is associated with an intracellular free Ca^2+^ rise. We hypothesize that ATP released from mast cells supplements the already known release of ATP from keratinocytes and, by acting on P2X receptors, it may serve as initial mediator of acupuncture-induced analgesia.

## 1. Introduction

 The treatment of specific points on the body surface by physical stimuli has been shown to affect various body functions including pain sensation [1, 2] and the cardiovascular system (see, e.g., [3]). Physical stimuli are also applied in traditional Chinese medicine (TCM) [4–6]: in needling acupuncture mechanical stress occurs during manipulation of lifting, thrusting, and twisting [5], and in moxibustion high temperatures exceeding 50°C are applied to the skin. More recently acupoints were treated with blue and red low-level laser light [7], termed laser acupuncture. 

Mast cells (MCs) play a significant role in the pathophysiology of many diseases including asthma and allergies, pulmonary fibrosis, and rheumatoid arthritis [8]. In addition to these deleterious activities, MCs are involved in protection from inflammation and help to maintain tissue homeostasis [9]. MCs are ubiquitous in the body, especially in interface, connective tissue, and mucous membranes. Skin is the main location of MCs [9], which makes the MCs easily accessible to the external physical stimuli. Recently, MCs were shown to participate in the mechanism of analgesia induced by needling acupuncture [5], moxibustion [10], and laser acupuncture [11]. In cellular biological studies, MCs can be activated by stretch and swelling, by application of heat or red laser irradiation that can be monitored morphologically and electrophysiologically [6, 12, 13]. Activation of transient receptor potential channel TRPV2 in the mast-cell membrane was suggested to be involved [6]. Once MCs are activated, their released mediators can be expected to activate sensory nerve fibers [14], and adenosine has been suggested as mediator of acupuncture-induced analgesia [15]. 

Extracellular nucleotides are important autocrine/paracrine mediators in various tissues. Increasing evidence suggests that extracellular ATP functions as a stress-responsive molecule, and mechanically induced ATP release is a cell-regulated process that could be observed in the absence of cell lysis [16]. In particular, mechanical stresses, such as stretch, shear, medium change, or osmotic stress, have been shown to evoke ATP release from various cell types [17]. Hypotonic shock represents experimentally convenient and frequently used surrogate of mechanical stress, with which it shares many common characteristics, including transient cytoskeleton reorganization, elevation of intracellular Ca^2+^ concentration ([Ca^2+^]_*i*_), and stimulation of other signaling pathways [18]. In some cell types, ATP release induced by mechanical stimulation correlates tightly with [Ca^2+^]_*i*_ elevation, suggesting the involvement of Ca^2+^-dependent exocytosis [19].

ATP release in response to mechanical stimulation from keratinocytes [15, 16] has been demonstrated, and activation of P2X2 and P2X2/3 receptors located on sensory nerve endings [15, 17] can be expected. Activation of purinergic signaling cascade in response to acupuncture has been suggested [20], and involvement of adenosine receptors in mediating local antinociceptive effects could be demonstrated in mice [15].

 Based on our previous studies on mechanical stress-, heat- and red laser light- induced degranulation and activation of TRPV2 channels of MCs  [6, 12, 13], we investigated whether these physical stimuli can liberate ATP from MCs. The human leukemia mast-cell line HMC-1 was used as a model system. The purpose of the study was to find a cellular basis for acupuncture effects; we suggest that ATP release from MCs might contribute to stimulation of P2X receptors [20–23] as an early step.

## 2. Methods

### 2.1. Cell Culture

 The HMC-1 was kindly provided by Dr. J. H. Butterfield (Mayo Clinic, Rochester, MN, USA). The cells were cultured as described previously [6]. In brief, cells were incubated in IMDM (Gibco, Invitrogen, Grand Island, NY, USA), supplemented with 2 mM L-glutamine, 25 mM HEPES, 10% (v/v) fetal bovine serum (Gibco, Invitrogen, Australia), and 1% penicillin and streptomycin (Gibco, Invitrogen, Grand Island, NY, USA), in a 95% humidity controlled incubator with 5% CO_2_ at 37°C.

### 2.2. Solutions and Reagents

Physiological solution (PS) contained (mM): 140 NaCl, 5 KCl, 1 CaCl_2_, 1 MgCl_2_, 10 D-glucose, and 10 HEPES, pH 7.4 (adjusted with NaOH). 50% hypotonic solution (HS) was prepared by adding equivalent distilled water to PS. Osmolarity of the solutions was checked with a freezing point osmometer (Micro Osmometer 3300, Advanced instruments Inc., Norwood, MA, USA). Ca^2+^-free solution was prepared by omitting CaCl_2_ and supplementing with 0.1 mM or 5 mM ethylene glycol-bis (*β*-aminoethyl ether)-N,N,N′,N′-tetraacetic acid (EGTA) to chelate trace Ca^2+^. 

100 mM N-Ethylmaleimide (NEM) (Sigma) was prepared in 100% ethanol and diluted into 200 *μ*M with IMDM medium before experiments. To apply NEM to cells, at the beginning of an experiment 50 *μ*L of upper supernatant in each sample was removed and replaced with 50 *μ*L IMDM containing 200 *μ*M NEM to obtain the final concentration of 100 *μ*M. Probenecid (Sigma) 250 mM stock solution was prepared in 1 M NaOH. Calcium Green-1 AM (Invitrogen) and Fura-2 AM (Invitrogen) were dissolved in 20% (w/v) Pluronic F-127 (Invitrogen). 25 mM 1, 2-Bis(2-aminophenoxy) ethane-N,N,N′,N′-tetraacetic acid tetrakis (acetoxymethyl ester (BAPTA-AM) (Sigma) was prepared with DMSO. All stock solutions were stored at −20°C and diluted into bath solution to working concentrations when used. DMSO and Pluronic F-127 were kept at less than 1% in all test solutions.

### 2.3. ATP Measurements

#### 2.3.1. Changes in ATP Release in Response to Mechanical Stimulation

In* in vitro* experiments, mechanical stimulation can be applied to cells in suspensions in different ways, for example, by hypotonic swelling, shear stress, or pressure stretch [24]. Here we exposed the HMC-1 cells to hypo-osmotic solution. ATP release in response to osmotic stress was measured with high temporal resolution using a flow-through filter chamber and open circuit perfusion system. Briefly, 1-2 mL of HMC-1 cell suspension (0.5–1.5 × 10^6^ cells/mL) was gently introduced, by gravity flow, into the polycarbonate filter chamber. It consisted of 25 mm or 13 mm diameter polycarbonate filter membrane with 1 *μ*m average pore size, which was mounted in appropriate polypropylene Swin-Lok Filter holder (Nucleopore, Whatman, Florham Park, NJ, USA). The chambers had internal volume of 700 *μ*L or 300 *μ*L, respectively. Cells were superfused with warm PS solution (37°C, in-line SF-28 heater, Warner Instrument Co., Hamden, CT, USA) at 1.3 mL/min. After an equilibration period in PS for 30–40 min, 50% hypotonic shock was applied by HS perfusion of the chamber (*t* = 0), and the perfusate was collected at 30 s or 60 s intervals with fraction collector Frac-100 (Pharmacia). ATP in the samples was quantified by luciferase-luciferin luminescence assay (ATP Assay Mix and ATP Assay Mix Dilution Buffer, Sigma-Aldrich Canada, Ltd). Luminescence was measured by Turner TD-20/20 luminometer (Turner Designs, Sunnyvale, CA, USA). 

#### 2.3.2. ATP Release in Response to Laser Irradiation

For low-level laser stimulation a CW laser of 656.7 nm (SB2007047, Shanghai University of TCM, China) was used with an output power of 35 mW. The diameter of the light spot was 0.4 cm.

HMC-1 cells were cultured in phenol-red-free IMDM medium. Cell density was adjusted to approximately 3.5 × 10^4^ cells/mL. Aliquots of 100 *μ*L cell suspensions were transferred into 1.5 mL Eppendorf tubes and placed in an incubator to equilibrate for 3 h. Following equilibration, some samples were exposed to red laser irradiation for 5 min as treated group and the remaining samples were the control group. 10 *μ*L rLuciferase-Luciferin (rL/L) reagents (Promega company, USA) were added into each sample and the luminescence was measured by the Luminometer (Promega company, USA). 

#### 2.3.3. ATP Release in Response to Heat

Samples were prepared as for the irradiation experiments. To determine temperature dependence of ATP release, the samples were kept at room temperature or placed into prewarmed water bath for 3 min at 42°C and 52°C, respectively. Before the measurement of the luminescence the heated samples were cooled down to room temperature for 1 min. 

ATP release was presented as changes of ATP concentration in the perfusate aliquots collected at different time points and expressed in nM/10^6^ cells. Calibration of luciferase-luciferin luminescence *versus* ATP standards was always performed with corresponding solutions used in the experiment. Moreover, all test compounds that were added to the extracellular solution during the ATP efflux experiments were also examined for their ability to directly interfere with luciferase bioluminescence. 

### 2.4. [Ca^2+^]_*i*_ Measurements

For [Ca^2+^]_*i*_ measurements in the experiments with mechanical stimulation cells were loaded (1 h, room temperature) with 10 *μ*M Fura-2-AM in physiological solution containing 0.02% Pluronic F127 and 2.5 mM Probenecid, followed by 30 min deesterification in PS-containing Probenecid. For fluorescence imaging, a Fura-2-loaded cells were introduced into an imaging/perfusion chamber (RC-20, volume 48 *μ*L) attached to a heated platform (P-5, Warner Instruments Co.) on the stage of an inverted microscope (Nikon TE300). A thin vacuum grease barrier was made at one end of the chamber, close to the perfusion outlet, to trap cells in the chamber and prevent their wash out during perfusion. The imaging chamber was perfused continuously with a warm solution (37°C) via an in-line heater (SF-28, Warner Instruments Co.) at ~0.5 mL/min. The cells were illuminated for 100 ms with alternate light wavelengths of 340 and 380 nm, using a high-pressure mercury lamp (100 W) via interference filters (Chroma Technology Corp., Brattleboro, VT, USA) mounted on a filter wheel (Sutter Lambda 10-C, Sutter Instrument Co., Novato, CA, USA) and a dichroic mirror (510/540 nm, Chroma Technology Corp.). Fluorescence images were recorded at 15 s intervals with a digital camera and stored for later analysis. Fura-2 measurements are presented as the fluorescence *F*_340_/*F*_380_ ratio. To chelate intracellular Ca^2+^, cells were preloaded with 25 *μ*M BAPTA-AM for 30 min at room temperature in PS. 

For [Ca^2+^]_*i*_ measurements in the experiments with laser irradiation and heat application, [Ca^2+^]_*i*_ measurements were performed as described previously [12]. In brief, HMC-1 cells grown on glass cover slips coated with poly-L-lysine (Sigma Chemical) were loaded with 4 *μ*M Calcium Green-1 AM in IMDM loading buffer for 1 h at room temperature. The loaded cells were superfused with PS. All solutions used in the fluorescence experiments contained 2.5 mM Probenecid. Irradiation experiments were performed at room temperature. In heating tests, 42°C and 52°C were controlled by a Temperature Control Device (PTC-20, NPI, Tamm, Germany). Photos were taken every minute. Images were digitized and averaged (five frames), background corrected, and analyzed by an image-processing system (Wasabi, Hamamatsu, Japan). Fluorescence intensities of individual cells in the field of view were determined by averaging the image intensities collected from regions of interest within each cell. 

In some experiments, Ca^2+^ influx from extracellular space was abolished by using nominally Ca^2+^-free extracellular medium containing 0.1 mM EGTA to chelate any trace of Ca^2+^.

### 2.5. Data Analysis

For data presentation and analysis, ORIGIN software (OriginLab, Northampton, MA, USA) was used. Data are expressed as mean ± SEM. The *n* values give the number of measurements obtained from different samples of cells; the *N* values the number of single cells analyzed in fluorescence measurements. Differences between sample means were evaluated by Student's *t*-test or Kruskal-Wallis test, and a *P* value <0.05 was considered to represent significant difference.

## 3. Results 

### 3.1. Stimulation by Mechanical Stress

**Figure 1:**
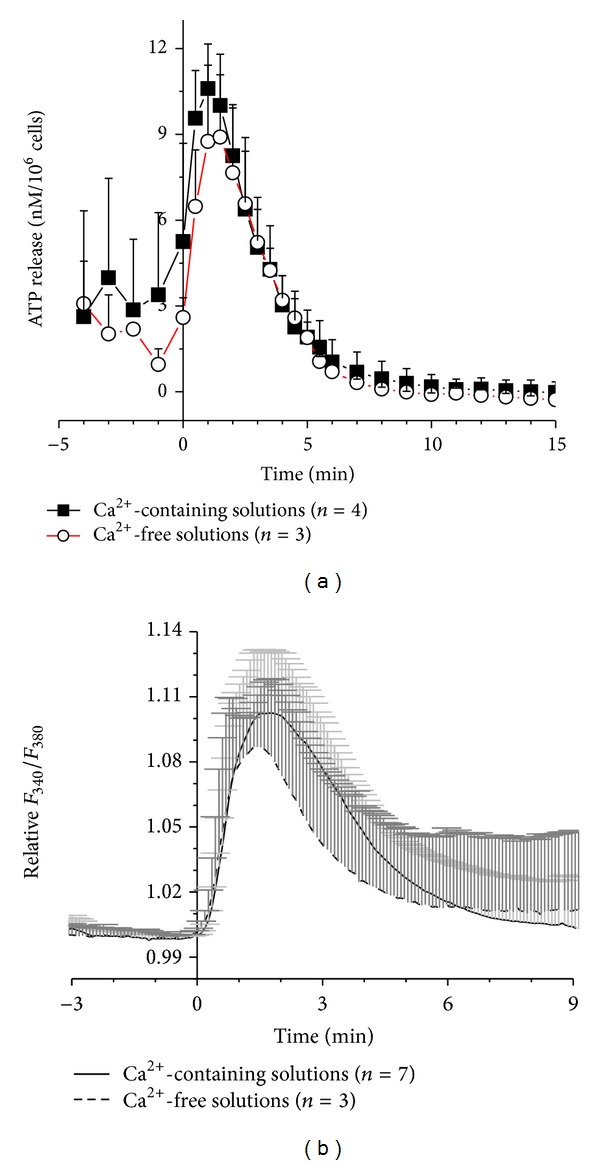
Time course of ATP release (a) and intracellular Ca^2+^ responses (b) induced by 50% hypotonic shock of HMC-1 cells in Ca^2+^-containing (filled squares (*n* = 4) or solid line (*n* = 7), resp.) and in Ca^2+^-free solutions (open circles (*n* = 3) of broken line (*n* = 3), resp.). Data represent averages of *n* measurements ± SEM. The respective curves in (a) and (b) are not significantly different on the basis of *P* > 0.05.

For mechanical stimulation of HMC-1 cells the perfusion solution in the flow-through filter chamber was changed at *t* = 0 to 50% hypotonic solution.  Figure 1(a) shows that ATP release transiently increased peaking after about 2 min and was followed by a decay that lasted approximately 10 min to a level that was even lower than that before stimulation. Short-term removal of extracellular Ca^2+^, that is, 3–5 min had no significant effect on the increase of ATP release by hypotonic shock (*P* = 0.4795 > 0.05, at 2 min). Fura-2 fluorescence measurements of [Ca^2+^]_*i*_ response showed a time course similar to the ATP release, that is, a peak of [Ca^2+^]_*i*_ at 1.5–2 min after stress application followed by a decay phase (Figure 1(b)). Similar to the ATP release, the [Ca^2+^]_*i*_ response was not significantly affected by removal of extracellular Ca^2+^ (*P* = 0.42503, at 2 min), suggesting that Ca^2+^ mobilization from intracellular stores plays a dominant role in this response. 

**Figure 2:**
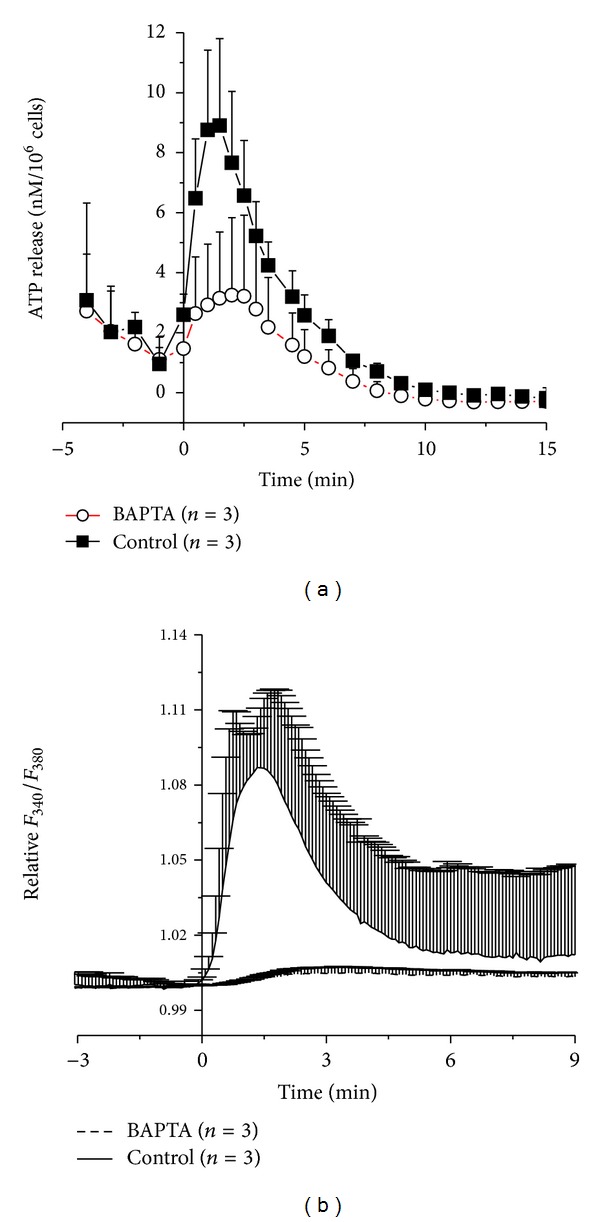
Time course of ATP release (a) and intracellular Ca^2+^ responses (b) induced by 50% hypotonic shock of HMC-1 cells before (filled square or solid line, resp.) and after treatment with BAPTA (open circles or broken line, resp.) in Ca^2+^-free solution. Data represent averages of 3 measurements each ± SEM. The respective curves in (a) and (b) are significantly different on the basis of *P* < 0.05.

To test the role of intracellular Ca^2+^ in hypotonic stress-induced ATP release, cells were pretreated with a Ca^2+^ chelator 25 *μ*M BAPTA-AM before they were subjected to hypotonic shock.  Figure 2(a) shows that in 50% hypotonic, Ca^2+^-free solution ATP release was significantly diminished in BAPTA-loaded cells compared to controls (*P* = 0.04953, at 2 min).  Figure 2(b) shows that also [Ca^2+^]_*i*_ response was almost completely abolished in these cells demonstrating a tight correlation between ATP release and [Ca^2+^]_*i*_ elevations induced by the hypotonic stress (*P* = 0.04953, at 2 min). 

### 3.2. Stimulation by Red Laser Light

**Figure 3:**
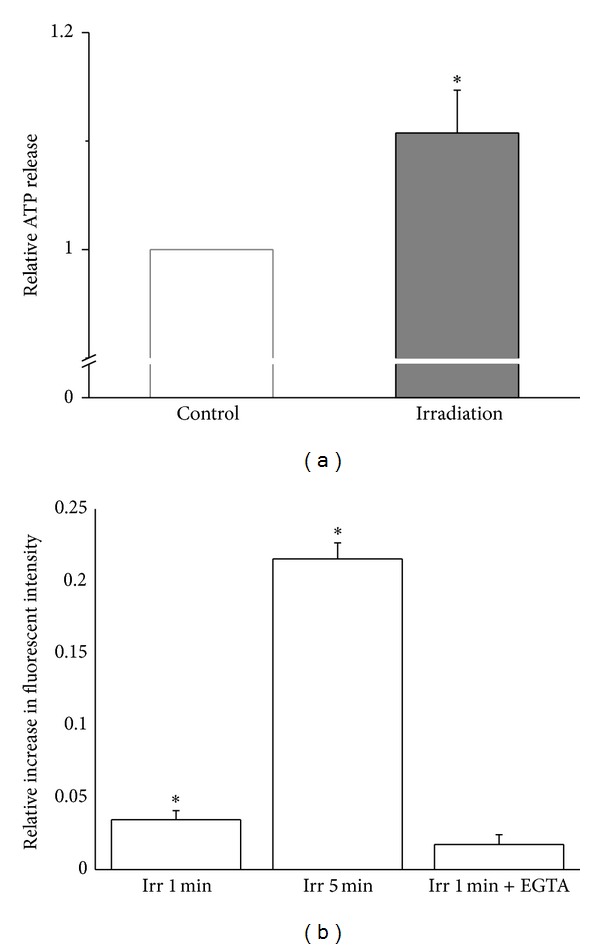
(a) Normalized ATP content in supernatant from untreated cells (Control) and of cells after having been treated for 5 min with red laser light (Irradiation). Data were normalized to the controls of the respective batch of cells and represent averages of 8 determinations (± SEM). One corresponds to 6.0 nM/10^6^ cells. (b) Relative increase in intracellular Ca^2+^ in response to 1 and 5 min of red laser light compared to control cells; measurements were performed with cells untreated (Irradiation) and cell treated for one min with EGTA (+EGTA). The data represent averages ±SEM (*N* = 15–17). *Significant difference compared to control.

To investigate the effect of red laser light on ATP release, HMC-1 cells were exposed to laser light of 656.7 nm at 35 mW. For cells that had been exposed for 5 min to laser light, the amount of released ATP was higher than in the controls by 10.7 ± 4.0% (*P* = 0.0299, *n* = 8). The concentration of released ATP for untreated control cells varied between different experiments from 4.5 nM to 8.7 nM.  Figure 3(a) shows averaged data for the light-induced ATP release normalized to the ATP level in control cells. 

Figure 3(b) illustrates that also [Ca^2+^]_*i*_ mobilization was induced by red laser irradiation. An increase of [Ca^2+^]_*i*_ by 3.5 ± 0.6% (*P* = 4.6 ×10^−6^, *N* = 17) and 21.5 ± 1.1% (*P* = 1.5 × 10^−11^, *N* = 15) appeared when HMC-1 cells were exposed to irradiation for 1 min and 5 min, respectively. The presence of 5 mM EGTA partially prevented [Ca^2+^]_*i*_ elevation induced by 1 min irradiation.

### 3.3. Stimulation by High Temperature

**Figure 4:**
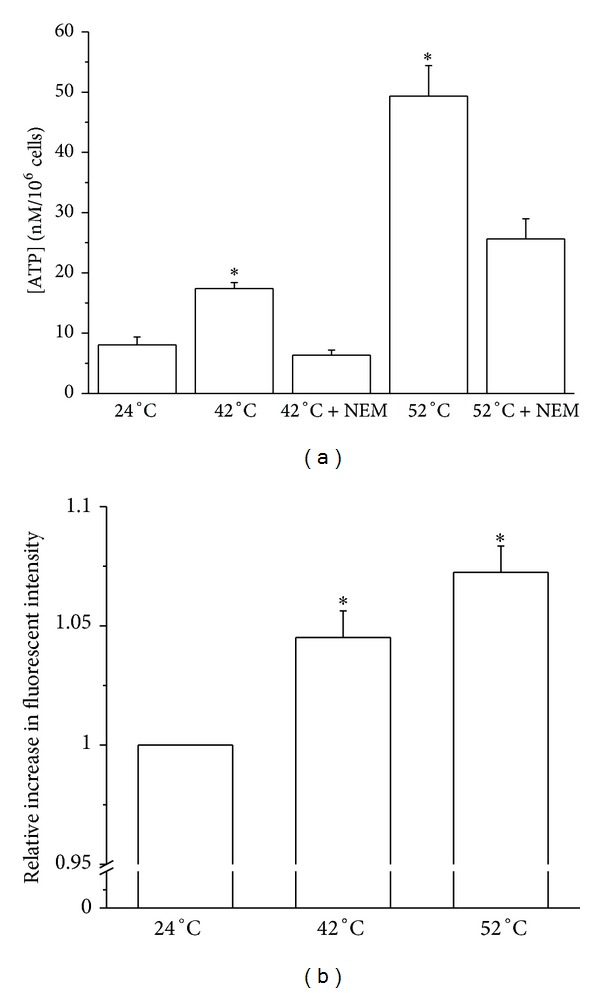
(a) ATP content in cell suspension after 3 min of incubation at different temperatures. Data represent averages ± SEM (*n* = 4–16). (b) Relative increase in fluorescent intensity of HMC-1 cells in response to higher temperatures. The data represent averages ± SEM (*N* = 13). *Significant difference compared to 24°C.

Figure 4(a) shows that incubation at 42°C increased the released ATP content from 8.0 ± 1.3 nM to 17.4 ± 1.0 nM (*P* = 0.0253), and an even more significant increase to 49.5 ± 5.0 nM (*P* = 1.8 × 10^−4^) was found at 52°C. In order to exclude that this response was only due to cell lysis, experiments were performed in solution containing 100 *μ*M NEM, an inhibitor of exocytosis [25]. In the presence of NEM the concentration of released ATP was reduced to 6.4 ± 0.8 nM at 42°C, which was close to the control value. At 52°C the ATP content dropped to 25.6 ± 3.3 nM. The result indicates that at least a large fraction of ATP release is mediated by exocytosis.

Similar to the mechanical and laser light stimuli, also heat could enhance [Ca^2+^]_*i*_ in HMC-1 cells. The relative fluorescent intensity increased by 4.5 ± 1.1% (*P* = 0.00112, *N* = 13) and 7.3 ± 1.1% (*P* = 9.0 ×10^−6^, *N* = 13) at 42°C and 52°C, respectively.

## 4. Discussion 

Recently we have shown that physical stimulation of MCs results in mast-cell degranulation [6, 12, 13], which forms an early step in acupuncture effects [5]. Here we have demonstrated that physical stimuli applied to MCs led to the release of ATP to the extracellular medium. These stimuli include hypo-osmotic stress with a transient stimulation reaching a maximum at about 2 min after the stimulation was initiated. This transient signal was paralleled by an increase in intracellular Ca^2+^ with nearly identical time course. While the intracellular [Ca^2+^]_*i*_ returned to the level before stimulation, the amount of ATP release dropped to even lower concentrations than before stimulation indicating partial depletion of ATP stores during the period of elevated release. Neither the release of ATP nor the increase in [Ca^2+^]_*i*_ was significantly affected if the experiments were performed in Ca^2+^-free medium; this observation suggests that the release of Ca^2+^ from intracellular stores might be involved in the process of ATP release. The hypothesis is supported by the finding that loading of the cells with the Ca^2+^ chelator BAPTA completely blocked the Ca^2+^ signal and strongly inhibited the ATP release. Similar observations were reported for hypotonic stress-induced ATP release from A549 cells, a model of human type 2 alveolar cells [18]. 

Irradiation with red laser light also stimulated ATP release. This increase in ATP release was also accompanied by an increase in [Ca^2+^]_*i*_. In contrast to hypo-osmotic stress, the 1 min irradiation-induced increase in [Ca^2+^]_*i*_ was partially reduced when the cells were kept for 1 min in Ca^2+^-free medium indicating that also extracellular Ca^2+^ contributes to this process. This agrees with our previous work, which demonstrated that red laser irradiation activated TRPV2 channels allowing Ca^2+^ uptake from extracellular space [6]. Basal ATP release from HMC-1 cells was observed when cells were kept at room temperature; however, its rate was significantly increased at elevated temperatures. The effect was more pronounced at 52°C than at 42°C. The temperature-dependent increase in ATP release cannot be attributed to cell lysis but rather to the combined effects of elevated temperature on exocytosis and Ca^2+^ homeostasis. Involvement of regulated exocytosis in the observed osmotic stress-induced and red laser light-induced ATP release is indicated by the accompanied increase of [Ca^2+^]_*i*_ and strong inhibition of the release by NEM. 

The physical stimuli applied in this investigation to the MCs are used in Chinese medicine to stimulate acupuncture points: mechanical stimulation during the needling, heat during moxibustion, and red light in laser acupuncture. Acupuncture and moxibustion have been demonstrated to be effective in analgesia [26]. Recently, pain relief by laser acupuncture has attracted attentions [11, 27, 28]. Purinergic signaling is known to participate in the mechanisms of pain sensation [29], and ATP is one of the main purinergic agonists in the purinergic system. Release of ATP from keratinocytes has also been suggested to be involved in acupuncture-dependent analgesia [20]. P2X3 homomeric and P2X2/3 heteromeric receptors are found predominantly in sensory nerve endings [30] and hence likely receptors for ATP released by physical stimulation of MCs and keratinocytes. Our results suggest that ATP release within the acupuncture point may be an initial step that may lead to stimulation of P2X3 and P2X2/3 receptors in peripheral nerve endings, which may account for acupuncture-induced analgesia [29]. For further elucidation of MC-neuron, interaction in response to the physical stimuli is under investigation. Since our data are obtained from *in vitro* experiments, further animal tests are needed for support. 

## Abbreviations

BAPTA-AM: 1,2-Bis(2-aminophenoxy) ethane-N,N,N′,N′-tetraacetic acid tetrakis (acetoxymethyl ester)
EGTA: Ethylene glycol-bis(2-aminoethylether)-N,N,N′,N′-tetraacetic acid
HS: Hypotonic solution
MCs: Mast cells
NEM: N-Ethylmaleimide
PS: Physiological solution
TRPV: Transient receptor potential channel (valinoidsensitive).
